# Interplay between Epigenetics, Expression of Estrogen Receptor- α, HER2/ERBB2 and Sensitivity of Triple Negative Breast Cancer Cells to Hormonal Therapy

**DOI:** 10.3390/cancers11010013

**Published:** 2018-12-21

**Authors:** Wafaa S Ramadan, Cijo George Vazhappilly, Ekram M Saleh, Varsha Menon, Aya M AlAzawi, Ahmed T El-Serafi, Wael Mansour, Raafat El-Awady

**Affiliations:** 1 Sharjah Institute for Medical Research, University of Sharjah, Sharjah 27272, UAE; wafaa.s.ramadan@hotmail.com (W.S.R.); cijo.vazhappilly@aurak.ac.ae (C.G.V.); vmenon@sharjah.ac.ae (V.M.); u00029765@sharjah.ac.ae (A.M.A.); aelserafy@sharjah.ac.ae (A.T.E.-S.); 2 College of Medicine, University of Sharjah, Sharjah 27272, UAE; 3 Cancer Biology Department, National Cancer Institute, Cairo University, Cairo 11796, Egypt; ekramsaleh@hotmail.com (E.M.S.); w.mansour@uke.de (W.M.); 4 Medical Biochemistry Department, Faculty of Medicine, Suez Canal University, Ismailia 41522, Egypt; 5 Radiobiology and Experimental Radiooncology laboratory, Center of Oncology, University Medical Center Hamburg, 20246 Hamburg, Germany; 6 College of Pharmacy, University of Sharjah, Sharjah 27272, UAE

**Keywords:** triple negative breast cancer, estrogen receptor alpha, human epidermal growth factor receptor-2, epigenetics, suberoylanilide hydroxamic acid, 2′-deoxy-5-azacytidine, tamoxifen

## Abstract

Triple negative breast cancer (TNBC) cells are resistant to hormonal/targeted therapies. This study aims to investigate epigenetic differences between TNBC and other types of breast cancer and the effect of epigenetic modulation on the response of TNBC cells to hormonal therapy. Thus, we investigated (i) the expression of different epigenetic markers, (ii) the effect of epigenetic modifying agents on the expression of ERα and HER2/ERBB2 and (iii) the effect on the response to tamoxifen in four breast cancer cell lines with different hormonal receptor status. Our results revealed a differential expression patterns of epigenetic markers in the four breast cancer cells. In TNBC cells, histone deacetylases (HDAC) 1 and 2 were less expressed, whereas HDACs 4 and 6 were overexpressed. Interestingly, treatment with epigenetic modifiers resulted in (i) a pronounced increase in the expression of ERα and HER2/ERBB2 along with (ii) an increase in the sensitivity of TNBC cells to tamoxifen. Collectively, this study indicates a different epigenetic background for TNBC cells, which represses the expression of ERα and HER2/ERBB2. Furthermore, we provide here the rationale for the use of epigenetic modifiers to enhance the response of TNBC to hormonal therapy through upregulation of ERα.

## 1. Introduction

Breast cancer (BC) is a heterogeneous disease, which is subclassified into categories depending on the expression of estrogen and progesterone receptors and the amplification of human epidermal growth factor receptor 2 (HER2/ERBB2) [1]. Triple negative breast cancer (TNBC) is one subtype that lacks the expression of steroid hormone receptors and HER2/ERBB2 gene amplification or protein overexpression [2]. TNBC is the most heterogeneous type and accounts for 15–20% of all breast cancer cases. It has poor clinical and pathological features and considered more aggressive compared to other subtypes of BC such as luminal A and B [3,4]. There are only limited treatment options for TNBC, owing to the lack of suitable targeted therapeutic drugs. TNBC generally shows partial response to the available drugs because of its aggressive phenotype. Due to the lack of expression of ERα and HER2/ERBB2 amplification/overexpression, TNBC cells normally do not respond to hormonal therapy using drugs such as tamoxifen [5,6,7,8,9]. Therefore, improving the response to hormonal therapy could be a potential promising approach in the management of TNBC.

Epigenetic modifications play a significant role in carcinogenesis and in the response to chemotherapy. This is effected through the regulation of genes involved in cell replication, tumor suppression, DNA repair, and apoptosis [10,11]. Targeting cellular epigenetic enzymes such as DNA methyltransferases (DNMT) and histone deacetylases (HDAC), modulates the chromatin structure and thereby affects gene expression [12,13]. The most established DNMT inhibitor is the cytidine analogue 2′-deoxy-5-azacytidine (5-aza-dc), which is approved by the Food and Drug Administration (FDA) for the treatment of myelodysplastic syndromes. The cytotoxic activity of 5-aza-dc is thought to be not only through its incorporation into DNA and RNA, but also by inhibiting the DNMT and targeting it to proteasomal degradation, thereby leading to DNA methylation reduction [14,15,16]. On the other hand, suberoylanilide hydroxamic acid (SAHA) is an HDAC inhibitor which targets class 1 and 2 HDACs. It is approved by FDA for the treatment of cutaneous T-cell lymphoma. It has a potent activity to inhibit tumor growth, induce differentiation, arrest cell cycle and promote apoptosis in variety of cancer cells, including BC [17,18,19]. 

In the present study, we investigated the effect of targeting the epigenetic modifying enzymes on the expression of ERα and HER2/ERBB2 and the possibility to sensitize BC cells to hormonal therapy. We report that manipulating the epigenetic machinery using 5-aza-dc and/or SAHA may provide an alternative treatment strategy to sensitize TNBC cells to hormonal therapy i.e. Tamoxifen. 

## 2. Results

### 2.1. Differential Expression of ERα, HER2/ERBB2, DNMT1 and HDACs in Breast Cancer Cells

**Figure 1:**
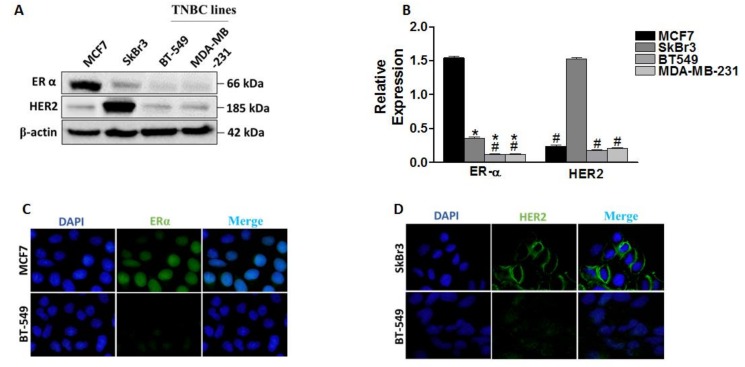
Differential expression of ERα and human epidermal growth factor receptor 2 (HER2/ERBB2) in breast cancer cells and their response to tamoxifen (TAM). (**A**) Western blot analysis of ERα and HER2/ERBB2 expression in MCF7, SkBr3, BT-549, and MDA-MB-231. Both proteins were visualized on the same blot. (**B**) Quantification of band intensities of the indicated proteins after normalization to β-actin. (**C**) Immunofluorescence detection of ERα in MCF7 and BT-549 (at 100× magnification). (**D**) Immunofluorescence detection of HER2 in SkBr3 and BT-549 (at 100× magnification). Shown are the means ± SEM of at least three independent experiments. * *p* < 0.05 versus MCF7 cells and # *p* < 0.05 versus SkBr3 cells. TNBC: triple negative breast cancer.

ERα and HER2/ERBB2 expressions contribute to hormonal therapy response in BC [20,21]. Here we analyzed the expression profile of these two markers in four BC cell lines (MCF7, SkBr3, BT-549 and MDA-MB-231), using western blot and immunofluorescence (Figure 1), and correlated their expression to the response to tamoxifen (TAM). Our results revealed differential expression levels of ERα and HER2/ERBB2 in the four cell lines (Figure 1A,B). We observed an elevated level of ERα in MCF7 and overexpression of HER2/ERBB2 in SkBr3 cells. This was further confirmed by immunofluorescence detection of ERα in MCF7/BT-549 and HER2/ERBB2 in SkBr3/BT-549 pairs (Figure 1C,D). As shown in Figure 1C, ERα signal was mainly observed in the nucleus in MCF7 cells, while it was absent in BT-549 cells. In addition, the signal of HER2/ERBB2 at the cell membrane of SkBr3 cells was higher compared to BT-549 cells (Figure 1D). 

Differences in the expression of ERα and HER2/ERBB2 were translated into differential responses to hormonal therapy with TAM as measured by Sulforhodamine B (SRB) assay (Table 1; Supplementary Figure S1A). ERα-positive MCF7 cells showed increased sensitivity to TAM with an IC50 of 6.8 ± 0.24 μM compared to the ERα-negative/low BC cell lines, which showed an IC50 more than 10 μM. Indeed, linear regression analysis revealed a significant correlation between baseline ERα expression and the sensitivity to TAM (r = −0.9654, *p* = 0.0346; Table 1 and Supplementary Figure S1B). In contrast with previous findings, no correlation was found between HER2/ERBB2 expression and sensitivity to TAM in our BC models (Table 1; Supplementary Figure S1C) [22]. 

**Figure 2:**
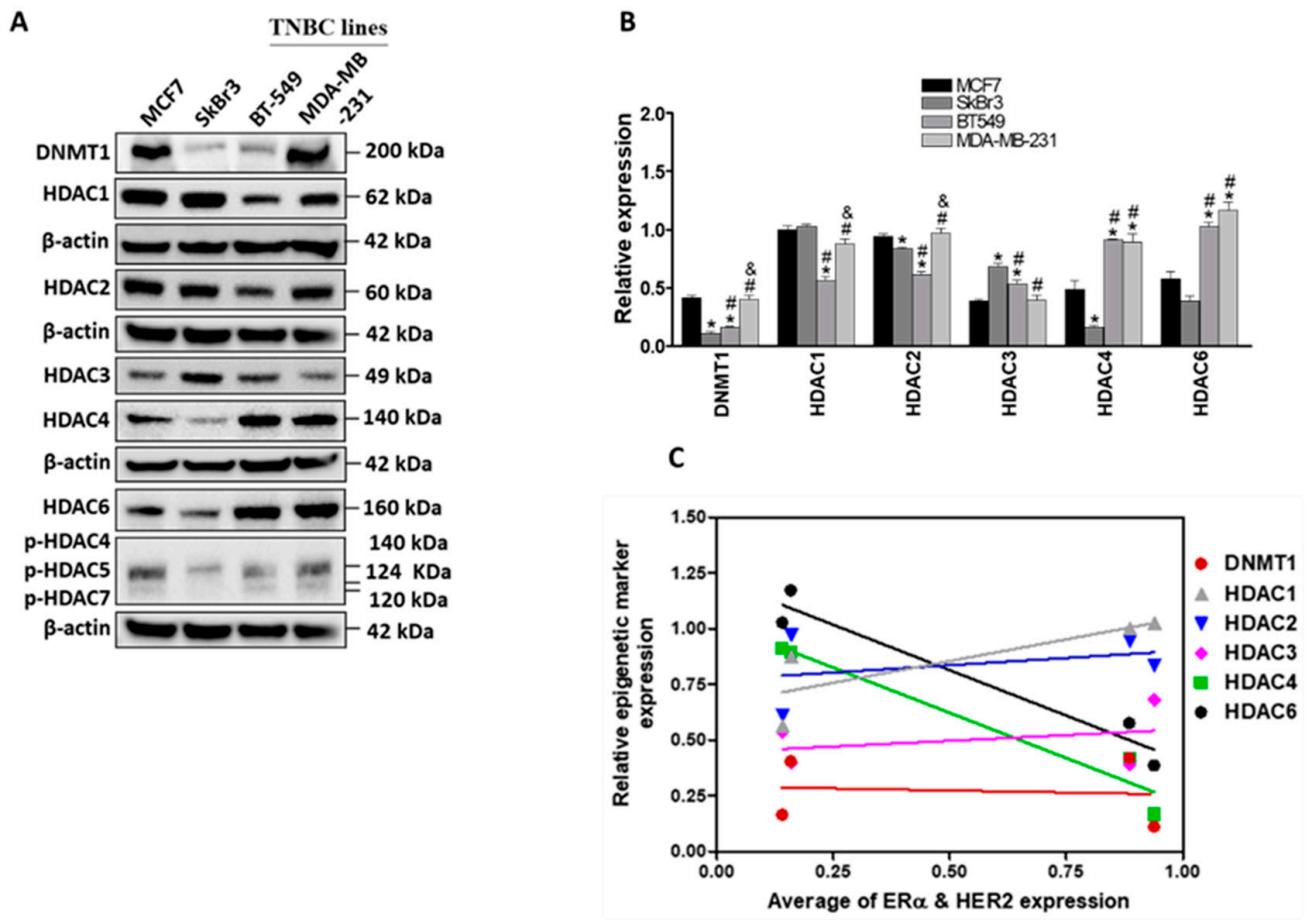
Differential expression of DNA methyltransferases (DNMT)1 and histone deacetylases (HDACs) in breast cancer cells. (**A**) Immunoblotting of DNMT1 and different HDACs in MCF7, SkBr3, BT-549 and MDA-MB-231. DNMT1 and HDAC1 were visualized on the same blot, HDAC2, HDAC6 and phospho- HDAC4,5 and 7 were visualized on another blot whereas HDAC3 and 4 were visualized on a third blot (**B**) Quantification of band intensities of the indicated proteins. Each protein visualized on a blot was normalized to the corresponding β-actin as a loading control. (**C**) Correlations between the expression levels of ERα and HER2 and the expression levels of DNMT1, HDAC1, HDAC2, HDAC3, HDAC4 and HDAC6 in the indicated cell lines. Shown are the means ± SEM of at least three independent experiments. * *p* < 0.05 versus MCF7 cells, # *p* < 0.05 versus SkBr3 cells and & *p* < 0.05 versus BT-549 cells. Abbreviation: ns, not significant.

Epigenetic regulations such as methylation and acetylation are main regulatory mechanisms for gene expression [10]. We next addressed the question whether the differential expression of ERα and HER2/ERBB2 in the indicated cell lines can be attributed to altered epigenetic regulations. To that end, the expression of different epigenetic markers (DNA methyltransferase 1, DNMT1, and histone deacetylases, HDACs) was analyzed in the four cancer cell lines (Figure 2). A differential expression of DNMT1, HDACs 1, 2, 3, 4, and 6 was observed in the examined cell lines (Figure 2A,B). Baseline levels of HDACs 1 and 2 were higher in growth-promoting receptor (ERα and HER2/ERBB2) positive cells (MCF7 and SkBr3), whereas HDACs 4 and 6 were higher in growth-promoting receptor negative cells (BT-549 and MDA-MB-231). Furthermore, the phosphorylation of HDACs 4, 5, and 7 was lower in SkBr3 cells than in the other three cell lines. Expression of DNMT1 was significantly higher in MCF7 and MDA-MB-231 cells than in the other two cell lines (Figure 2B). Linear regression analysis showed a negative correlation between the expression of growth-promoting receptors and the baseline levels of both HDAC4 (r = −0.9731, *p* = 0.0269) and HDAC6 (r = −0.9711, *p* = 0.0289) (Figure 2C and Table 2). However, no significant correlation was observed between the expression of other epigenetic markers (DNMT1, HDACs 1, 2, and 3) and the level of ERα and HER2/ERBB2 in the four cell lines (Figure 2C and Table 2). 

These results reveal some differences in the expression of different epigenetic markers in the growth-promoting receptors positive cells with HDAC4 and HDAC6 being negative regulators of ERα/HER2 expression.

### 2.2. Effect of SAHA and/or 5-aza-dc on the Expression of Epigenetic Modulators

Chromatin remodeling agents such as SAHA and 5-aza-dc can alter chromatin structure which may modify the expression status of many genes, leading to change in the response to cancer therapy. To address this issue, we investigated the effect of SAHA and 5-aza-dc on the expression levels of the epigenetic modifiers DNMT1 and HDACs. Firstly, the sensitivity of the four BC cell lines to SAHA (Supplementary Figure S2A) and 5-aza-dc (Supplementary Figure S2B) was measured by colony formation assay and the IC50 values were determined for each cell line (Supplementary Figure S2C). Results revealed that the growth-promoting receptor positive cell lines (MCF7 and SkBr3) were about 3-fold more sensitive to SAHA treatment than the TNBC cell lines, whereas the sensitivity to 5-aza-dc was independent on the expression level of these receptors (Supplementary Figure S2B).

**Figure 3:**
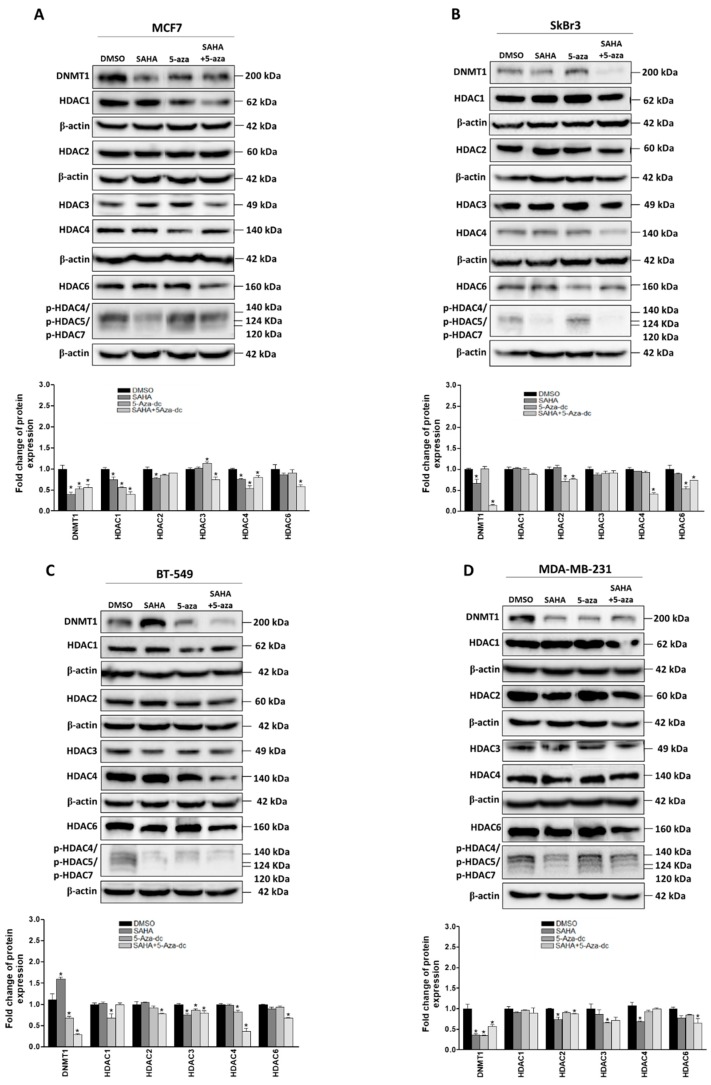
Effect of suberoylanilide hydroxamic acid (SAHA) and 5-aza-dc on the expression of epigenetic markers in breast cancer cells. Analysis of DNMT1, HDAC1, 2, 3, 4, 6, and p-HDAC 4/5/7 proteins upon treatment with IC50 concentrations of SAHA for 72 h or/and 5-aza-dc for 96 h in (**A**) MCF7, (**B**) SkBr3, (**C**) BT-549, and (**D**) MDA-MB-231. DNMT1 and HDAC1 were visualized on the same blot, HDAC2, HDAC6 and phospho- HDAC4,5 and 7 were visualized on another blot whereas HDAC3 and 4 were visualized on a third blot. Bar graphs showing the relative fold change of DNMT1 and HDACs after normalization to β-actin expression and DMSO treatment. The level of each protein was normalized to the corresponding β-actin from the same blot. Shown are the means ± SEM of at least three independent experiments. * *p* < 0.05 versus DMSO group.

Next, the effect of SAHA and/or 5-aza-dc on the expression levels of DNMT1 and different HDACs was measured by western blot after treating the cells with the respective IC50 concentrations (Figure 3A–D). We found that the expression of DNMT1 was significantly reduced upon treatment with SAHA in MCF7, SkBr3 and MDA-MB-231 cells (Figure 3A,B,D). Notably, SAHA treatment resulted in an unexpected increase in DNMT1 expression in BT549 cells (Figure 3C). 5-aza-dc treatment decreased the expression of DNMT1 in MCF7, BT-549, and MDA-MB-231 (Figure 3A,C,D) but not in SkBr3 cells (Figure 3B). Strikingly, combined SAHA and 5-aza-dc treatment decreased more efficiently the expression of DNMT1 in all four cell lines including BT549 cells, which showed an increase in the expression of DNMT1 upon treatment with SAHA (Figure 3C). On the other hand, the effect of the aforementioned drugs either individually or combined showed a differential effect on the expression profile of HDACs and their phosphorylation in the four cell lines. In MCF7 cells, a reduction in HDAC1 expression was reported upon treatment with SAHA and 5-aza-dc either combined or individually. On the other hand, a reduction in HDAC 2 expression was observed in MDA-MB-231 cells after SAHA and combined treatments (Figure 3A,D). Moreover, the expression of HDAC3 was down-regulated by SAHA, 5-aza-dc and their combination in BT549 cells but only by 5-aza-dc in MDA-MB-231 cells. HDAC4 expression was significantly reduced after 5-aza-dc treatment in MCF7 and BT-549 cells (Figure 3A,C). Noteworthy, HDAC4 and HDAC6 were down-regulated upon combined 5-aza-dc and SAHA treatment more profoundly in TNBC cell lines. In addition, the phosphorylation levels of HDACs 4, 5, and 7 were reduced in all cell lines upon treatment with either SAHA alone or combined with 5-aza-dc. Together, these data reveal that the indicated epigenetic modifiers can generally alter the expression of DNMT1 and/or HDACs, which may result in modulating the chromatin structure and subsequently change gene expression.

### 2.3. Effect of SAHA and/or 5-aza-dc on the Expression Level of ERα

**Figure 4:**
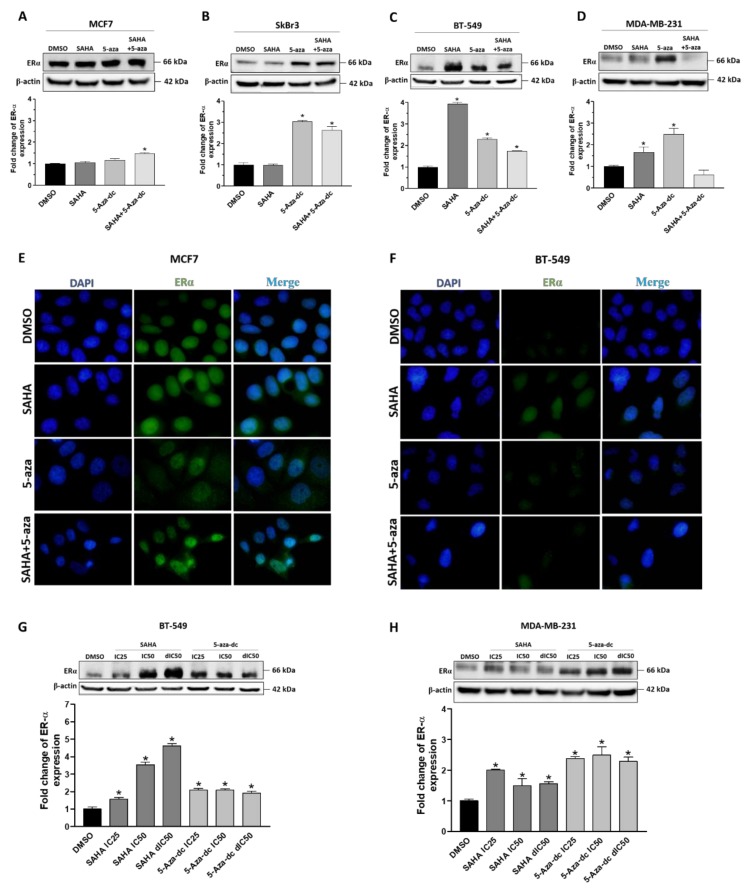
Effect of SAHA, 5-aza-dc and their combination on the expression of ERα in breast cancer cells. (**A**–**D**) Upper panels: Western blot analysis of ERα expression in (**A**) MCF7, (**B**) SkBr3, (**C**) BT549 and (**D**) MDA-MB-231 cells treated with the IC50 concentrations of SAHA and/or 5-aza-dc. Lower panel: Bar graphs showing relative fold changes of ERα bands after quantification and normalization to β-actin expression and DMSO treatment. (**E**,**F**) Representative micrographs (at 100× magnification) of immunofluorescence staining of ERα (green) and 4′,6-diamidino-2-phenylindole (DAPI)(blue) in (**E**) MCF7 and (**F**) BT-549 cells treated with SAHA and/or 5-aza-dc. (**G**,**F**) Western blots of ERα expression in (**G**) BT-549 and (**H**) MDA-MB-231 cells treated with IC25, IC50, and double IC50 (dIC50) concentrations of SAHA or 5-aza-dc. Shown are the means ± SEM of at least three independent experiments. * *p* < 0.05 versus DMSO group.

We next sought to investigate whether modifying the epigenome might increase the expression of ERα. Thus, we examined the effect of SAHA and 5-aza-dc on the expression of ERα using western blot. Generally, our results revealed that treatment of the four cell lines with SAHA, 5-aza-dc or their combination showed different effects on the expression level of ERα (Figure 4; Supplementary Figure S3). An increase in ERα expression was shown upon SAHA treatment in BT-549 and MDA-MB-231 cells compared to MCF7 and SkBr3 cells (Figure 4A–D). 5-aza-dc treatment, on the other hand, enhanced the level of ERα in SkBr3, BT-549 and MDA-MB-231 and to lesser extent in MCF7 cells. Combined SAHA and 5-aza-dc treatment upregulated ERα expression in MCF7, SkBr3, and BT-549 cells (Figure 4A–C). Immunofluorescence analyses confirmed the upregulation of ERα by SAHA, 5-aza-dc and combined treatment in MCF7 and BT-549 cells (Figure 4E,F). Of note, the ERα expression was observed in the nucleus and cytoplasm compartments in both cell lines. SAHA resulted in a concentration-dependent increase in the level of ERα only in BT-549 cells (Figure 4G). On the other hand, 5-aza-dc upregulated the expression of ERα in both SkBr3 and MDA-MB-231 cells independent of drug concentration (Supplementary Figure S3B; Figure 4H). In contrast, this increase in the expression of ERα was not observed in MCF7 cells upon treatment with SAHA or 5-aza-dc (Figure 4A; Supplementary Figure S3A). These results were indeed further recapitulated using immunofluorescence staining (Supplementary Figure S3C,D). Collectively, these findings suggest that the expression of ERα can be modulated by SAHA or/and 5-aza-dc especially in TNBC cells.

### 2.4. Effect of SAHA and/or 5-aza-dc on the Expression Level of HER2/ERBB2

**Figure 5:**
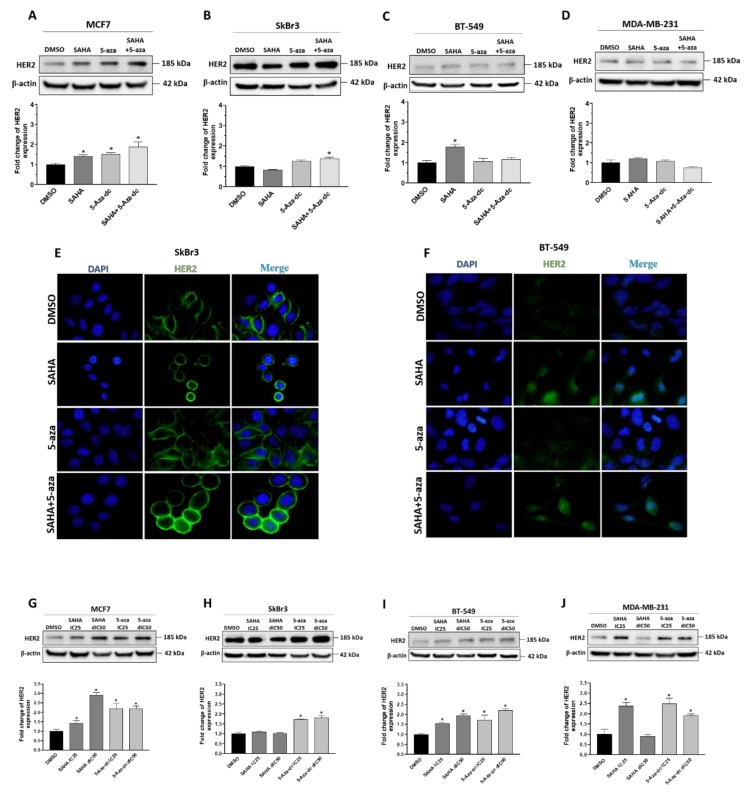
Effect of SAHA, 5-aza-dc and their combination on the expression of HER2/ERBB2 in breast cancer cells. (**A**–**D**) Western blot analysis of HER2/ERBB2 expression in (**A**) MCF7, (**B**) SkBr3, (**C**) BT549, and (**D**) MDA-MB-231 cells after treatment with the IC50 concentrations of SAHA and/or 5-aza-dc. β-actin was used as a loading control. Lower panels: Bar graphs showing relative fold changes of HER2/ERBB2 expression after quantification and normalization to β-actin expression and DMSO treatment. (**E**,**F**) Representative micrographs of HER2/ERBB2 (green) and DAPI (blue) fluorescent signals at 100× magnification in (**E**) SkBr3 and (**F**) BT-549 cells treated with SAHA and/or 5-aza-dc. (**G**–**J**) Upper panels: Western blots for HER2/ERBB2 expression in (**G**) MCF7, (**H**) SkBr3, (**I**) BT549, and (**J**) MDA-MB-231 cells after treatment with IC25 or double IC50 (dIC50) concentrations of SAHA or 5-aza-dc. Lower panels: Bar graphs showing relative fold changes of HER2 after normalization to β-actin and DMSO treatment. Shown are the means ± SEM of at least three independent experiments. * *p* < 0.05 versus DMSO group.

HER2/ERBB2 expression was previously reported to affect the response to TAM [22]. Therefore, the effect of SAHA and/or 5-aza-dc treatment on the expression of HER2/ERBB2 was investigated by western blot and immunofluorescence. The two epigenetic modifying agents have differentially modulated the expression of HER2/ERBB2 in the four cell lines (Figure 5; Supplementary Figure S4). IC50 concentrations of SAHA enhanced the expression of HER2/ERBB2 in MCF7 and BT549 cells (Figure 5A–D). 5-aza alone or in combination with SAHA significantly increased the expression of HER2/ERBB2 in MCF7 cells with minor or even no effect on the other three cell lines. In addition, the IC25 and double IC50 concentrations of SAHA or 5-aza-dc more prominently upregulated the expression of HER2/ERBB2 in all cells (Figure 5G–J). HER2/ERBB2 upregulation was further confirmed in SkBr3 and BT-549 cells using immunofluorescence analysis (Figure 5E,F; Supplementary Figure S4A,B). Of note, HER2/ERBB2 was detected at the membrane in SkBr3 cells whereas, it was found both in cytoplasm and nucleus in BT-549 cells. These data demonstrate that SAHA and/or 5-aza treatment alters the expression of HER2/ERBB2 in BC cells.

### 2.5. Effect of SAHA and/or 5-aza-dc on the Response of Breast Cancer Cells to Hormonal Therapy

Next, we sought to monitor the effect of ERα/HER2 upregulation—mediated by epigenetic modifiers—on the response of BC cells to hormonal therapy i.e., TAM. Therefore, colony formation assay was used to measure the sensitivity of the four BC cell lines to different concentrations (0.01–10 μM) of TAM in combination with respective IC25 and IC50 concentrations of SAHA and/or 5-aza-dc (Table 3 and Supplementary Figure S5). MCF7 cells were found to be the most sensitive (~2-fold) to TAM with an IC50 of 6.67 ± 0.23 µM, while the other cell lines showed IC50 more than 10 µM. Importantly, the addition of IC25 concentrations of SAHA significantly increased the sensitivity of MDA-MB-231 (~800 fold) but showed no effect on MCF7, BT549 and SkBr3 cells. Addition of IC50 concentrations of SAHA increased the response of MCF7 and both TNBC cells to TAM compared to SkBr3 cells. Similarly, the addition of IC50 or even the IC25 concentrations of 5-aza-dc increased the sensitivity of TAM in all cell lines. A similar increase in TAM sensitivity was reported in all cell lines upon combining with both SAHA and 5-aza-dc using IC25 or IC50 concentrations. Altogether, these results demonstrate that manipulating the epigenome could be an efficient strategy to enhance the response of TAM-resistant cells. 

### 2.6. Effect of SAHA and/or 5-aza-dc on Cell Cycle Distribution and Apoptosis in Breast Cancer Cells

**Figure 6:**
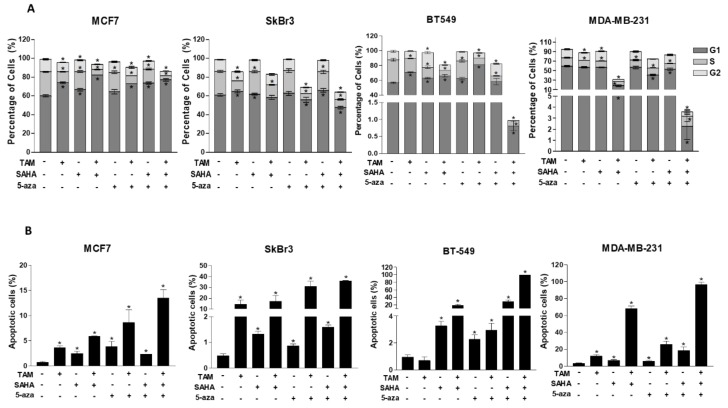
Effect of TAM, SAHA, 5-aza-dc and their combination on cell cycle distribution. (**A**) Bar graphs showing the percentage of cells in each cell cycle phase after treatment of MCF7, SkBr3, BT-549 and MDA-MB-231 cells with IC50 concentrations of SAHA and/or 5-aza-dc and their combination with the IC50 concentration of TAM. (**B**) Percentage of sub-G1 cells (apoptotic cells) for each cell line is indicated. Shown are the means ± SEM of at least three independent experiments. * *p* < 0.05 versus DMSO group.

Having established the profound effect of both SAHA and/or 5-aza-dc as sensitizing agents to TAM, we therefore investigated whether these epigenetic modifiers may have cellular effects other than regulation of ERα expression. Thus, the effect of SAHA or/and 5-aza-dc on cell cycle progression as well as apoptosis was analyzed in the four cell lines using flow cytometry (Figure 6 and Supplementary Figure S6). Propidium iodide (PI) staining of the four cell lines treated with the respective IC50 concentrations of SAHA showed (i) a significantly increased accumulation of TNBC cells at G2 phase (BT-549: 1.7-fold, *p* < 0.0001; MDA-MB-231: 1.2-fold, *p* = 0.0049) and (ii) an increase in G1 phase fraction in MCF7 and BT549 cells. On the other hand, 5-aza-dc induced a G1-arrest exclusively in BT-549 cells (1.1-fold). The effect of combined treatment of TAM with SAHA or 5-aza-dc resulted in a G1 arrest in both MCF7 and BT-549 cells.

Apoptosis—indicated by the percentage of sub-G1 cells—was induced significantly in the four cell lines after treatment with SAHA, 5-aza and their combination (Figure 6B). Importantly, the combination of these epigenetic modifiers with TAM further increased apoptosis more apparently in TNBC cells (Figure 6B). The expression of different apoptotic markers after treatment with SAHA and/or 5-aza-dc was examined in the four cell lines using western blot (Supplementary Figure S7). On the one hand, IC50 concentrations of SAHA reduced the expression of the apoptotic agonist BID in MCF7, SkBr3 and MDA-MB-231 and enhanced caspase 3 cleavage in BT-549 cells (Supplementary Figure S7C). On the other hand, 5-aza-dc treatment increased (i) the expression of p53 in all cell lines (ii) caspase 9 cleavage in MCF7 cells and (iii) caspase 3 cleavage in the other three cell lines. Noteworthy, SAHA or 5-aza-dc treatment changed the expression level of caspase 3, 8 and 9 in MDA-MB-231 cells more than the other cell lines (Supplementary Figure S7D). The combination of SAHA and 5-aza-dc reduced (i) the expression of bcl-xl in MCF7 cells and (ii) the expression of BID in MCF7 and TNBC cells. These data indicate that both SAHA and 5-aza-dc exert their effect additional through apoptosis induction, however through different mechanisms. While, SAHA induces apoptosis probably through downregulating the anti-apoptotic factors bcl-xl and BID, 5-aza-dc does this through stimulating the expression of p53 and increasing the processing of caspases 3 and 9.

## 3. Discussion

Aberrant epigenetic modifications have been shown to play an important role in BC tumorigenesis, progression, and treatment response, through impacting DNA repair, cell cycle, apoptosis and hormonal regulation [23,24]. Accordingly, modulating DNA methylation and histone acetylation might be a promising therapeutic strategy in breast cancer. In the current study, we report different expression patterns of epigenetic markers such as DNMT1 and different HDACs in four BC cell lines that differ basically in the expression of ERα and HER2/ERBB2 ([25] and results reported herein). This is consistent with the clinical studies that showed differential expression of HDACs 1, 2, and 3 in BC patients with different hormonal status [26]. DNMT1 was unexpectedly low in the TNBC cell line ‘BT-549’ compared to that in MCF7 and MDA-MB-231 cells, because DNA methylation is partly involved in ERα and HER2/ERBB2 gene silencing [27]. Indeed, several other methyltransferases such as DNMT3b were shown to be responsible for silencing ERα and HER2/ERBB2 genes [28]. Further, we report here that HDAC4 and 6 are stronger regulators of ERα and HER2/ERBB2 expression than other HDACs. HDAC4/6 were found to be highly expressed in BT549 and MDA-MB-231 cells, which have low levels of ERα and HER2/ERBB2. Vice versa, HDAC 4 and 6 expressions were low in MCF7 and SkBr3 cells that have high expression of ERα and overexpression of HER2/ERBB2, respectively. These results are consistent with a previous observation in ERα-negative cells where HDAC 6 overexpression was described [29]. We report here that ERα but not HER2/ERBB2 expression level can predict TAM sensitivity in the four cell lines. Previously, HER2 overexpression was found to associate with TAM resistance in BC, properly through a crosstalk with ERα signaling pathway [22,30]. The discrepancy between these studies and ours could be attributed to other cytotoxic effects of TAM such as induction of apoptosis and cell cycle modifications which might overcome the estrogenic (anti-TAM) effects induced by HER2 expression. 

An interplay between DNA methylation and histone deacetylation to modify the expression level of some genes has been previously reported by Jones et al., where HDAC was shown to be recruited at the promoter area of the target gene after attachment of 5-methylcytosine binding protein [31]. This crosstalk was the foundation to investigate the effect of combining 5-aza-dc and SAHA on the expression level of different genes in our panel of cell lines. In the present study, SAHA and 5-aza-dc showed differential effects on the expression of DNMT1 and HDACs. In consistent with previous reports [32], the expression of HDAC 1 or 2 was not modified in ERα-negative cell lines upon treatment with SAHA. However, SAHA treatment decreases the expression of (i) HDAC1 in MCF7, (ii) HDAC2 in MDA-MB-231, and (iii) HDACs 3 in BT-549 cells. This is most likely due to modulation of the chromatin structure by SAHA that would affect the normal transcription level of some genes [33]. Interestingly, a reduction in the expression of HDACs 4 and 6 was detected upon treatment with 5-aza-dc and its combination with SAHA. This indeed supports the interplay between DNA methylation and histone acetylation in chromatin remodeling [34,35]. 

The reported modulation of ERα/HER2 expression by SAHA and/or 5-aza-dc, especially in TNBC cells, suggests that epigenetic modifications might have a role in regulating the expression of these receptors. Based on our findings, we hypothesize that (i) the decreased HDACs levels/activities mediated by SAHA stimulates H3 and/or H4 acetylation at ERα promoter and (ii) the decreased level/activity of DNMT by 5-aza-dc compromises the methylation at certain CpG sites within ERα promoter. These epigenetic modifications allow an open chromatin state at the promoter of the ERα/HER2 genes, thus enhancing the expression of both receptors. This indeed in line with previously published data showing that the HDAC inhibitor Trichostatin A and 5-aza-dc modulate chromatin structure at ER promoter in TNBC cells MDA-MB-231 through increasing H3 and H4 acetylation and partial demethylation of CpG islands respectively [36]. The concentration-dependent upregulation of ERα by SAHA in TNBC cells further supports this finding. However, the effect of combining SAHA and 5-aza-dc on ERα expression was found to be similar to the effect observed for single treatment. This can be explained by the existence of a ceiling effect for ERα expression that can be achieved by a single treatment and cannot be exceeded upon combined treatment. In line with this, Pryzbylkowski et al. demonstrated a reduction in ERα mRNA stability upon combination treatment with 5-aza-dc and Trichostatin-A through reducing cytoplasmic levels of the RNA-binding protein HuR that is responsible for ERα mRNA stabilization [37]. 

The upregulation of ERα expression observed after SAHA and/or 5-aza-dc tempted us to use these epigenetic modifiers to sensitize TNBC cells to hormonal therapy such as TAM. Strikingly, we report an enhanced response of BC cells to TAM upon combination with either of the aforementioned epigenetic modifier. This could be partly but not exclusively attributed to ERα upregulation by SAHA or/and 5-aza-dc. SAHA and/or 5-aza-dc increased the expression of ERα by 4–5 folds, yet they increased the sensitivity to TAM by several hundred times. Previously, several reports suggested that HDAC inhibitors (SAHA and Trichostatin-A) and demethylating agents (zebularine and 5-aza-dc) induce apoptosis in different cancer types including BC [38,39,40,41]. Consistently, we report that SAHA or/and 5-aza-dc and their combination with TAM enhanced apoptosis and arrested the cells in G1 or G2, importantly with greater impact on TNBC cells.

**Figure 7:**
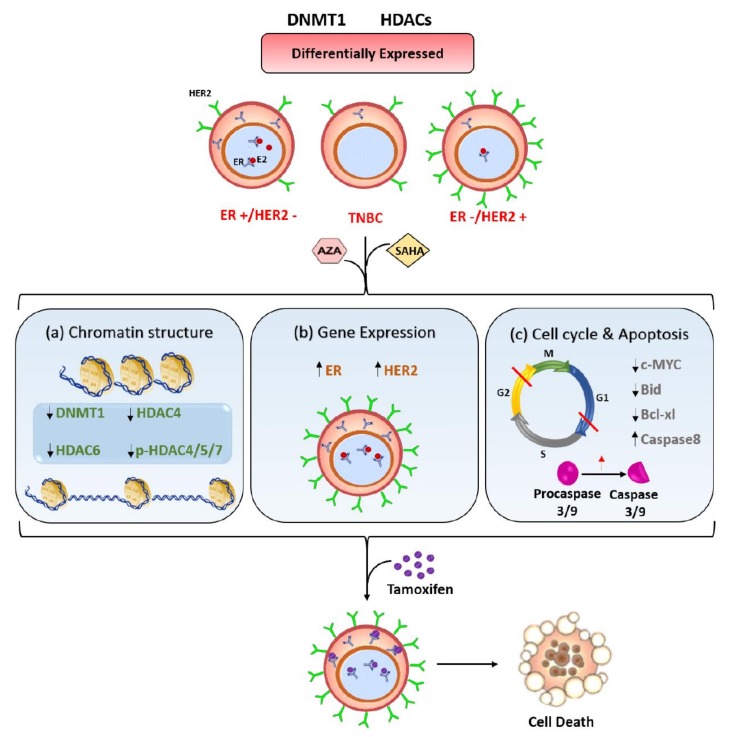
Model for the effect of SAHA and 5-aza-dc on the response of TNBC cells to TAM. BC cells with different hormonal status vary in the expression pattern of DNMT1 and different HDACs. Epigenetic modifiers such as SAHA and 5-aza-dc result in (**a**) modifying the chromatin structure by reducing DNMT1 expression and inhibiting the activity and phosphorylation of different HDACs, (**b**) upregulating the expression of ERα and HER2/ERBB2 and (**c**) arresting the cell cycle in G1 and G2 phases and inducing apoptosis. These changes consequently increase the response of TNBC cells to TAM.

Collectively, we propose here a mechanistic model for targeting TNBC cells using epigenetic modifiers to enhance TAM sensitivity (Figure 7). SAHA and 5-aza-dc induce chromatin modifications mainly by changing the expression of DNMT1 and HDACs 4 and 6. Subsequently, these modifications upregulate the expression of ERα and HER2/ERBB2, as well as, induce cell cycle arrest and apoptosis. These cellular changes eventually enhance the response of TNBC cells to TAM. 

## 4. Materials and Methods

### 4.1. Cell Lines and Culture Conditions

Four human BC cell lines (MCF-7, BT-549, SkBr3 and MDA-MB-231) were employed in the present study. All cell lines were maintained in RPMI or DMEM medium supplemented with 10% fetal bovine serum and 1% penicillin/streptomycin (Sigma Aldrich, St. Louis, MO, USA) in a 37 °C humidified incubator and an atmosphere of 5% CO_2_. The expression status of the hormone receptors ER, PR and Her2/ErBB2 in these four cell lines are shown in Table 4.

### 4.2. Colony Formation Assay

Sensitivity to tamoxifen, SAHA and 5-AZA was tested using colony formation assay as previously described [42]. Briefly, cells were cultured at different numbers (50–1200) in T25 cm^2^ culture flasks. After 24 h, cells were treated with either 0.01–10 µM of SAHA for 72 h or 0.01–10 µM of 5-aza-dc (Sigma Aldrich) for 96 h. In combination treatments, cells were treated with 0.01–10 µM of tamoxifen (Sigma Aldrich) alone or in combination with IC25 or IC50 concentrations of SAHA. For combination with 5-aza-dc, the treatment started with 5-aza-dc for 24 h followed by the addition of tamoxifen and/or SAHA. DMSO (Sigma-Aldrich) was used as a control. DMSO concentration was <0.01% in all experiments. At the end of the treatment period, the drug-containing medium was replaced with drug-free medium and cells were further incubated. Colonies were then fixed with 70% of ethanol for 30 min and stained with 1% crystal violet (Sigma-Aldrich) for 5 min at room temperature. The number of colonies at each treatment was counted for the calculation of both platting efficiency and surviving fraction. The IC50 values were calculated by sigmoidal curve fitting models using Graph Pad Prism 3 software (GraphPad Software, San Diego, CA, USA). 

### 4.3. Sulforhodamine B (SRB) Assay

Anti-proliferative effect of tamoxifen was investigated using sulforhodamine B assay (Sigma-Aldrich), according to manufacturer’s protocol [43]. Briefly, cells were cultured at density of 7000 cells/well in a 96-well plate format. After 24 h, cells were treated with 1–160 µM of tamoxifen for further 72 h. Thereafter, cells were fixed with 50% trichloroacetic acid for 1 h at 4 °C. Plates were washed 9 times with water and stained with 0.4% SRB for 30 min at room temperature. After washing with 1% acetic acid, the dye was solubilized in 200 μL of 10 mM Tris base for 10 min. The optical density (OD) was measured at 492 nm in a microplate reader Varioskan™ Flash (Thermo Fisher Scientific, Waltham, MA, USA)

### 4.4. Western Blot

Western blot was conducted as previously described [44]. Cells were washed with 1× phosphate buffered saline (PBS) (Sigma Aldrich) and harvested with lysis buffer containing 20% SDS, glycerol, 1 M Tris (pH 6.8) and protease inhibitor cocktail (Sigma-Aldrich). Equal amounts of total protein (30 µg) were separated on 12% SDS polyacrylamide gel and transblotted onto polyvinylidene difluoride (PVDF) membrane (Biorad, Hercules, CA, USA). The membranes were blocked with 5% non-fat dried milk in 1X TBS-Tween 20 and then incubated with primary antibodies at a dilution of 1:1000. The following antibodies were used: ERα (D8H8) Rabbit mAb #8644, HER2/ErbB2 Rabbit pAb #2242, HDAC1 (10E2) Mouse mAb #5356, HDAC2 (3F3) Mouse mAb #5113, HDAC3 (7G6C5) Mouse mAb #3949, HDAC4 (D15C3) Rabbit mAb #7628, HDAC6 (D2E5) Rabbit mAb #7558, Phospho-HDAC4 (Ser246)/HDAC5 (Ser259)/HDAC7 (Ser155) (D27B5) Rabbit mAb #3443, DNMT1 (D63A6) XP^®^ Rabbit mAb #5032, BID Antibody (Human Specific) Rabbit pAb #2002, Bcl-xL (54H6) Rabbit mAb #2764, c-Myc (D84C12) Rabbit mAb #5605, Caspase-3 (8G10) Rabbit mAb #9665, Caspase-8 (1C12) Mouse mAb #9746 and Caspase-9 (C9) Mouse mAb #9508 (purchased from Cell Signaling Technology, Danvers, MA, USA), p53 (DO-1) Mouse mAb #sc126 (Santa Cruz Biotechnology, Dallas, TX, USA) and Anti-β-Actin Clone AC-15 Mouse mAB #A5441 (Sigma Aldrich) for overnight at 4 °C. The secondary antibodies Anti-rabbit IgG, HRP-linked Antibody #7074 and Anti-mouse IgG, HRP-linked Antibody #7076 (Cell Signaling Technology) were prepared at dilution of 1:2000 and incubated with membrane at room temperature for 1 h. The membranes were then washed 3 times with 1× TBS-T. Chemiluminescence was detected by ECL method (Thermo Fisher Scientific) and developed by ChemiDoc^TM^ imaging system (Biorad). Quantification of bands was analyzed by Image Lab^TM^ software (Biorad).

### 4.5. Immunofluorescence

Immunofluorescence detection of ERα and HER2/ERBB2 was performed as previously described [45]. Cells were cultured on coverslips and at the end of the treatment period, cells were fixed with 4% formaldehyde, permeabilized with 0.5% Triton-X (Sigma-Aldrich) and blocked with 4% BSA in humidifying chamber for 20 min. Coverslips were then incubated with ERα (D8H8) Rabbit mAb #8644 or HER2/ErbB2 (29D8) Rabbit mAb #2165 (Cell Signaling Technology) at a dilution of 1:250 for 1 h at room temperature, followed by an addition of fluorescine iso-thiocyanate (FITC)-conjugated secondary antibody #4412 (Cell Signaling Technology) at a 1:500 dilution for 45 min at room temperature. After washing, cells were mounted on glass slides with prolong antifade reagent with DAPI (Cell Signaling Technology). Cells were then visualized by BX51P fluorescence microscope (Olympus, Tokyo, Japan) at 100× magnification. 

### 4.6. Cell Cycle Analysis

Effects on cell cycle were analyzed using flow cytometry as previously described [46]. At the end of treatment period, cells were harvested, washed with 1× PBS and then fixed in 70% ethanol for 24 h at 4 °C. The fixed cells were washed 2 times with 1× PBS and incubated in 1× PBS containing RNAase (100 µg/mL) for 30 min at 37 °C. Fixed cells were subsequently stained with propidium iodide (50 µg/mL) and analyzed using FACScan flow cytometry (Becton Dickenson, San Jose, CA, USA). DNA histograms and data analysis, including the calculation of sub-G1 population were performed with FlowJo V.10 software (Tree Star, Inc, Ashland, OR, USA) as previously described [47]. 

### 4.7. Statistical Analysis

All experiments were carried out in triplicate and repeated at least 3 times. Data are expressed as means ± SEM. Statistical analysis was performed by unpaired student’s *t*-test using Graph Pad Prism software (GraphPad Software). *p* < 0.05 was considered statistically significant. The linear regression and Pearson’s correlation coefficient (r) was calculated using Graph Pad Prism software (GraphPad Software)

## 5. Conclusions

Collectively, we report that SAHA and 5-aza-dc either individually or in combination (i) modulate the expression of some epigenetic regulators i.e., DNMT1 and HDACs, (ii) increase the expression of ERα especially in ER-negative cells, and eventually (iii) sensitize breast cancer cells to TAM treatment. These findings need to be confirmed using animal models and subsequent clinical trials. Clinically, our data provides the proof-of-concept of using epigenetic modifiers to sensitize TNBC cells to hormonal therapy.

## Figures and Tables

**Table 1:** IC50 values of TAM, relative expression level of ERα and HER2/ERBB2 and their correlation to the sensitivity of the four cell lines to TAM.

Cell Line	MCF7	SkBr3	BT549	MDA-MB-231
**TAM IC50 (µM)**	6.8 ± 2.4	12.1 ± 0.89	11.9 ± 0.56	13.8 ± 0.1
**Relative ERα expression**	1.54 ± 0.02	0.35 ± 0.02	0.11 ± 0.01	0.11 ± 0.01
**Correlation**	r = −0.09654, *p* = 0.0346			
(TAM IC50 versus Relative ERα expression)				
**Relative HER2 expression**	0.23 ± 0.03	1.53 ± 0.02	0.17 ± 0.01	0.21 ± 0.005
**Correlation**	r = −0.1877, *p* = 0.8123			
(TAM IC50 versus Relative HER2 expression)				

Shown are the means ± SEM of at least three independent experiments. Indicated are the r values (Pearson’s correlation coefficient) with the corresponding *p* values.

**Table 2:** Statistical parameters of correlation analysis between the expression levels of ERα and HER2 and the expression levels of epigenetic markers.

Correlation		
Epigenetic Markers	r Values	*p* Values
**DNMT1**	−0.1003	ns
**HDAC1**	0.8099	ns
**HDAC2**	0.3509	ns
**HDAC3**	0.3238	ns
**HDAC4**	−0.9731	0.0269
**HDAC6**	−0.9711	0.0289

Indicated are the r values (Pearson’s correlation coefficient) with the corresponding *p* values.

**Table 3:** IC50 values of SAHA and/or 5-aza-dc and their combination with TAM against four breast cancer cell lines.

	IC50 (μM) ± SEM			
Cell Line	MCF7	SkBr3	BT-549	MDA-MB-231
SAHA	0.600 ± 0.034	0.570 ± 0.042	1.500 ± 0.089	1.580 ± 0.12
5-aza-dc	0.040 ± 0.0028	0.080 ± 0.0062	1.040 ± 0.074	0.026 ± 0.0011
SAHA + 5-aza-dc	0.107 ± 0.0312	0.0284 ± 0.0056	0.0097 ± 0.0021	0.0082 ± 0.0024
Tamoxifen	6.4481 ± 0.23	> 10	> 10	>10
Tam + IC25 SAHA	6.3241 ± 0.31	> 10	> 10	0.0126 ± 0.00078
Tam + IC50 SAHA	0.5420 ± 0.014	> 10	0.0022 ± 0.0001	0.0025 ± 0.0001
Tam + IC25 5-aza-dc	0.0117 ± 0.0009	0.0059 ± 0.00024	0.0022 ± 0.0001	0.0030 ± 0.0002
Tam + IC50 5-aza-dc	0.0031± 0.00013	0.0027 ± 0.00008	0.0021 ± 0.00023	0.0025 ± 0.0001
Tam + IC25 SAHA + IC25 5-aza-dc	0.0087± 0.00035	0.0022 ± 0.0001	0.0020 ± 0.00008	0.0021 ± 0.00009
Tam + IC50 SAHA + IC50 5-aza-dc	0.0024 ± 0.00011	0.0022 ± 0.00009	0.0020 ± 0.00015	0.0021 ± 0.00007

Shown are the means ± SEM of at least three independent experiments.

**Table 4:** Expression status of ERα, PR, and HER2/ERBB2 in four breast cancer cell lines [25].

Cell Line	Estrogen Receptorα (ERα)	Progesterone Receptor (PR)	HER2/ERBB2 (Overexpression)
MCF7	Yes	Yes	No
SkBr3	No	No	Yes
BT-549	No	No	No
MDA-MB-231	No	No	No

## Supplementary Materials

The following are available online at http://www.mdpi.com/2072-6694/11/1/13/s1, Figure S1: The response of breast cancer cells to TAM and the correlation analysis with ERα and HER2/ERBB2 expression, Figure S2: Effect of SAHA and 5-aza-dc on survival of breast cancer cells, Figure S3: Effect of SAHA and 5-aza-dc on the expression of ERα in breast cancer cells, Figure S4: Effect of SAHA and 5-aza-dc on the expression of HER2/ERBB2 in breast cancer cells, Figure S5: Effect of SAHA and/or 5-aza-dc on the sensitivity of breast cancer cells to TAM, Figure S6: Effect of TAM, SAHA, 5-aza-dc and their combination on cell cycle distribution, Figure S7: Effect of SAHA, 5-aza-dc and their combination on apoptosis in breast cancer cells.
