# Supplementary material for: Interplay between Epigenetics, Expression of Estrogen Receptor- α, HER2/ERBB2 and Sensitivity of Triple Negative Breast Cancer Cells to Hormonal Therapy
Source: Cancers (Basel). 2018 Dec 21;11(1):13. doi: 10.3390/cancers11010013 (PMC6356506; doi:10.3390/cancers11010013)
Supplement: Supplementary file 1 [file cancers-11-00013-s001.pdf]

Supplementary Materials

Interplay between Epigenetics, Expression of Estrogen Receptor- α, HER2/ERBB2 and Sensitivity of Triple Negative Breast Cancer Cells to Hormonal Therapy

Wafaa S Ramadan, Cijo George Vazhappilly, Ekram M Saleh, Varsha Menon, Aya M AlAzawi, Ahmed T El-Serafi, Wael Mansour and Raafat El-Awady

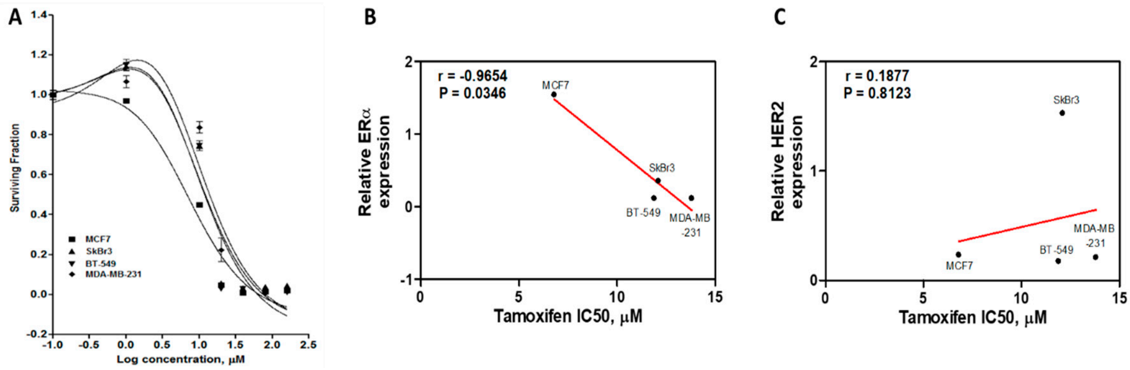

Figure S1. The response of breast cancer cells to TAM and the correlation analysis with ERα and HER2/ERBB2 expression: (**A**) Surviving fractions of MCF7, SkBr3, BT-549 and MDA-MB-231after treatment with increasing concentrations of TAM. −1 on the X-axis corresponds to 0 µM. (**B**,**C**) Correlation analysis between TAM IC50 and the relative expression level of ERα (**B**) and HER2/ERBB2 (**C**) in breast cancer cell lines. The r values indicate Pearson's correlation coefficient with corresponding p value.

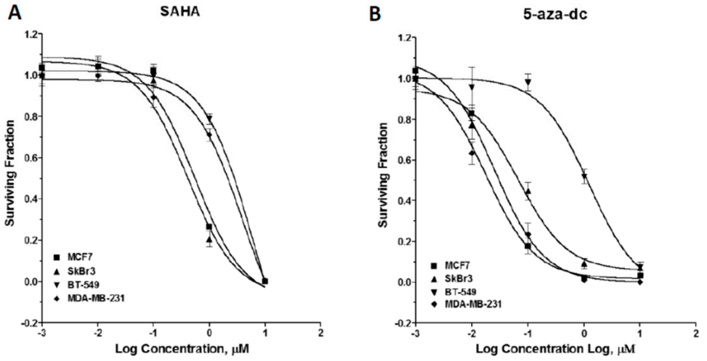

C

	IC50 (µM) ± SEM	
**Cell line**	**SAHA**	**5-aza-dc**
**MCF7**	0.600±0.034	0.040±0.0028
**SkBr3**	0.570±0.042	0.080±0.0062
**BT-549**	1.500±0.089	1.040±0.074
**MDA-MB-231**	1.580±0.12	0.026±0.0011

Figure S2. Effect of SAHA and 5-aza-dc on survival of breast cancer cells. Surviving fractions of the indicated breast cancer cell lines were measured by colony formation assay after treatment with increasing concentrations of (**A**) SAHA or (**B**) 5-aza-dc. (**C**) IC50 of SAHA and 5-aza-dc in the indicated cells were calculated by best fitting curve method. Shown are the means ± SEM of at least three independent experiments.

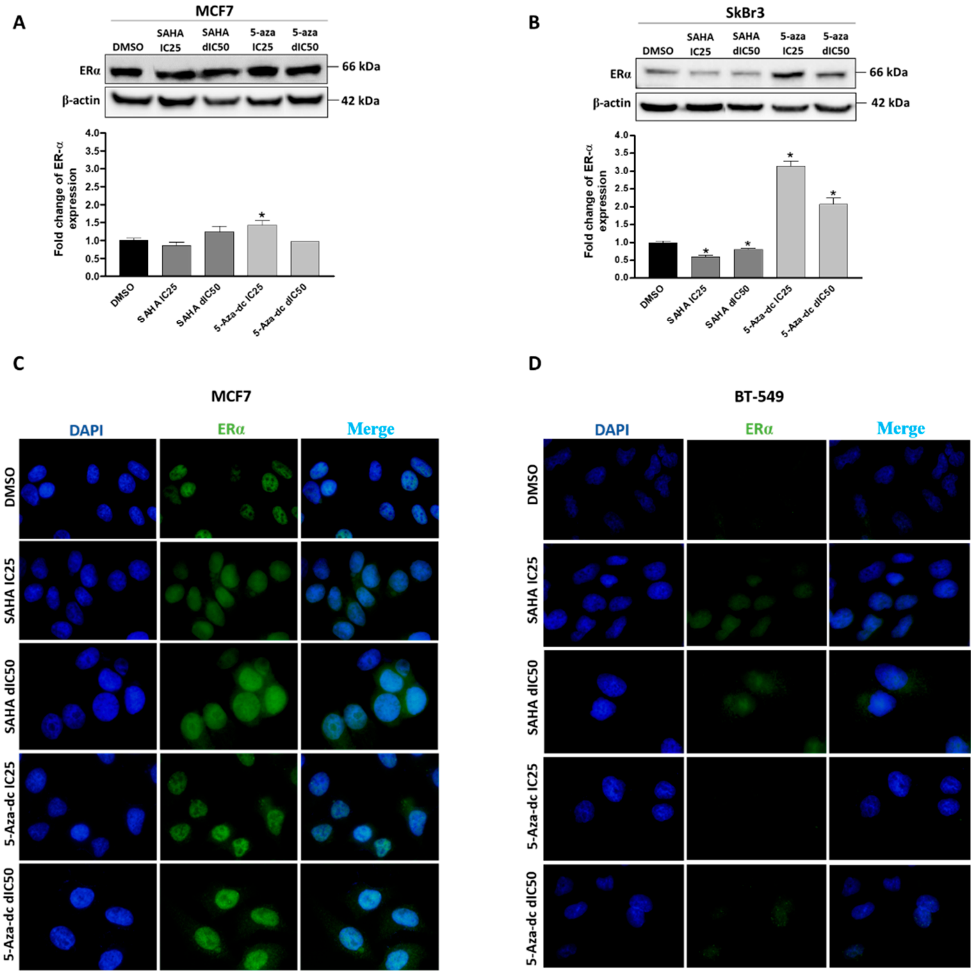

Figure S3. Effect of SAHA and 5-aza-dc on the expression of ERα in breast cancer cells. (**A**,**B**) upper panels: Western blot analysis of ERα expression in (**A**) MCF7 and (**B**) SkBr3 cells after treatment with IC25 or double IC50 (dIC50) concentrations of SAHA or 5-aza-dc. Lower panels: Bar graphs showing relative fold changes of ERα after quantification and normalization to β-actin and DMSO treatment. (**C**,**D**) Representative images (at 100× magnification) for immunofluorescence staining of ERα (green) and DAPI (blue) in (**C**) MCF7 and (**D**) BT-549 cells treated with IC25 or double IC50 (dIC50) concentrations of SAHA or 5-aza-dc. Shown are the means ± SEM of at least three independent experiments. * $p < 0.05$ vs. DMSO group.

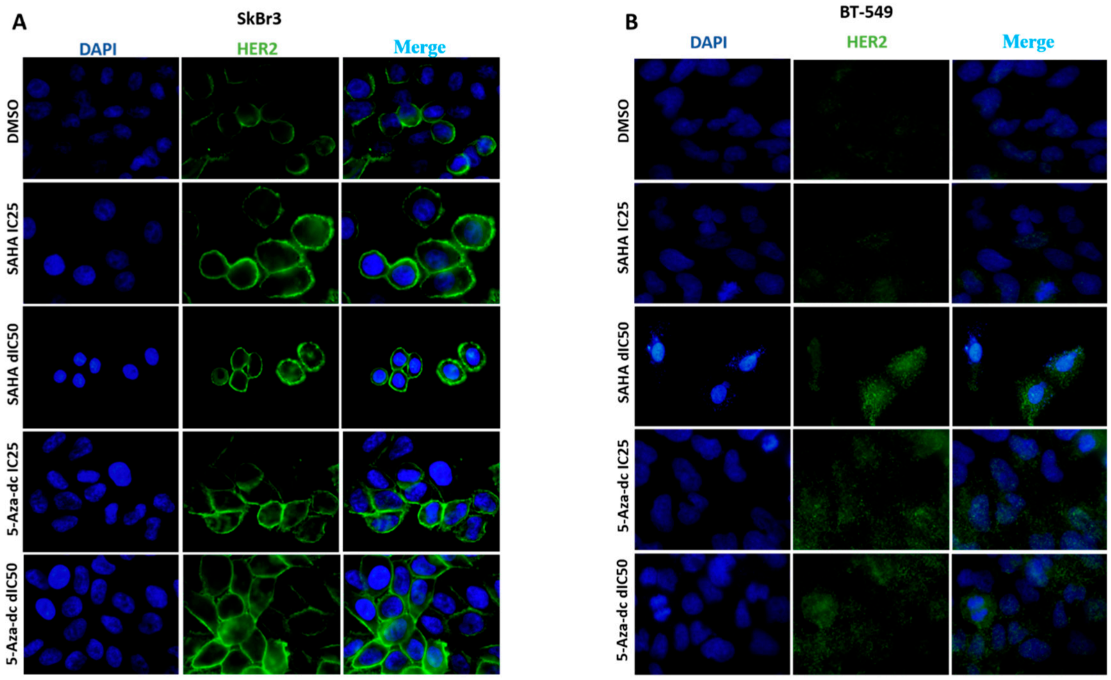

Figure S4. Effect of SAHA and 5-aza-dc on the expression of HER2/ERBB2 in breast cancer cells. (**A**,**B**) Representative micrographs (at 100× magnification) for immunofluorescence staining of HER2 (green) and DAPI (blue) in (**A**) SkBr3 and (**B**) BT-549 cells after treatment with IC25 or double IC50 (dIC50) concentrations of SAHA or 5-aza-dc.

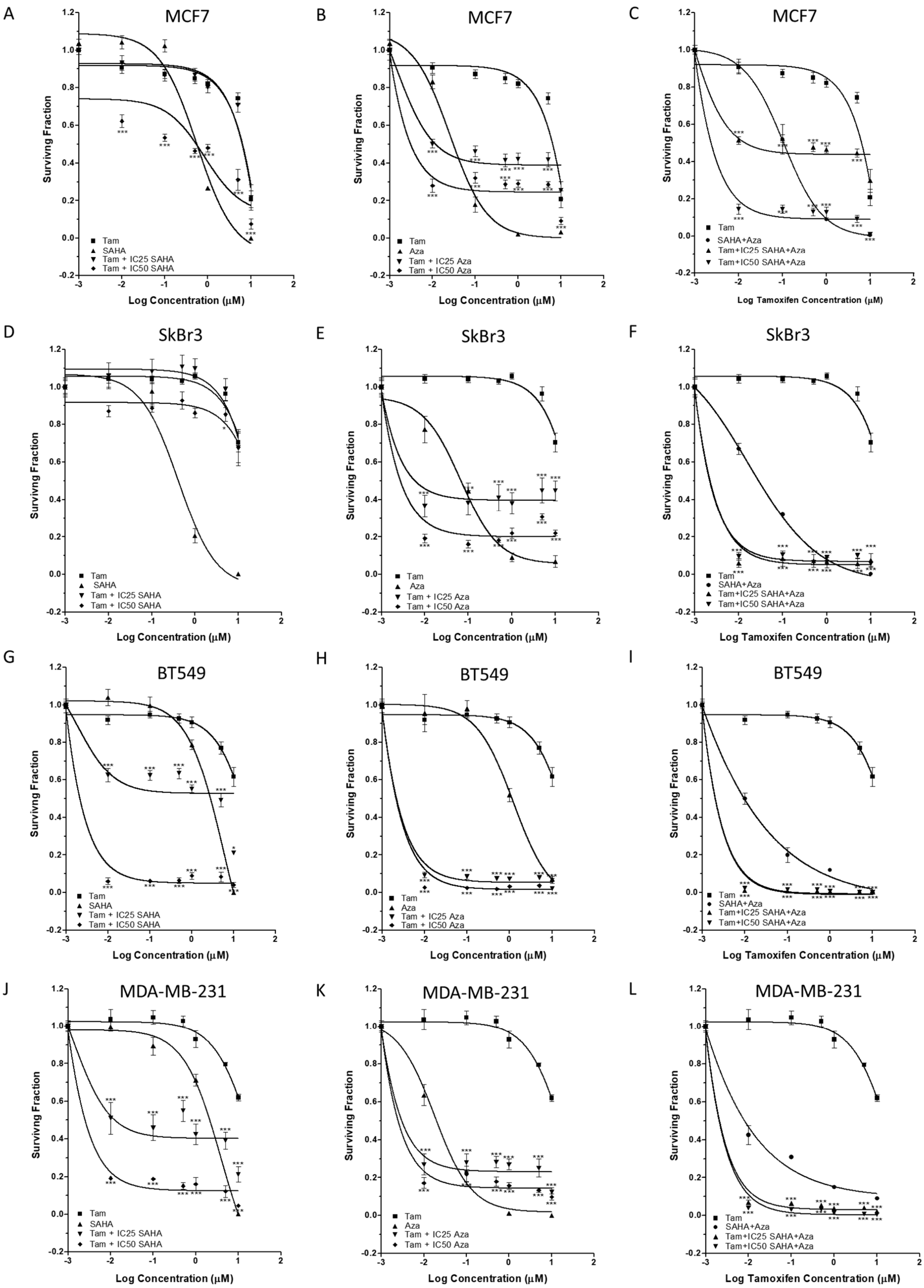

Figure S5. Effect of SAHA and/or 5-aza-dc on the sensitivity of breast cancer cells to TAM. Surviving fractions were calculated by colony formation assay in MCF7 (**A**–**C**), SkBr3 (**D**–**F**), BT-549 (**G**–**I**) and MDA-MB-231 (**J**–**L**) cells after treatment with increasing concentrations of TAM in combination with either IC25 or IC50 of SAHA and/or 5-aza-dc. −3 on the X-axis corresponds to 0 μM. Shown are the means ± SEM of at least three independent experiments. * $p < 0.05$, ** $p < 0.01$, *** $p < 0.001$ vs. TAM group.

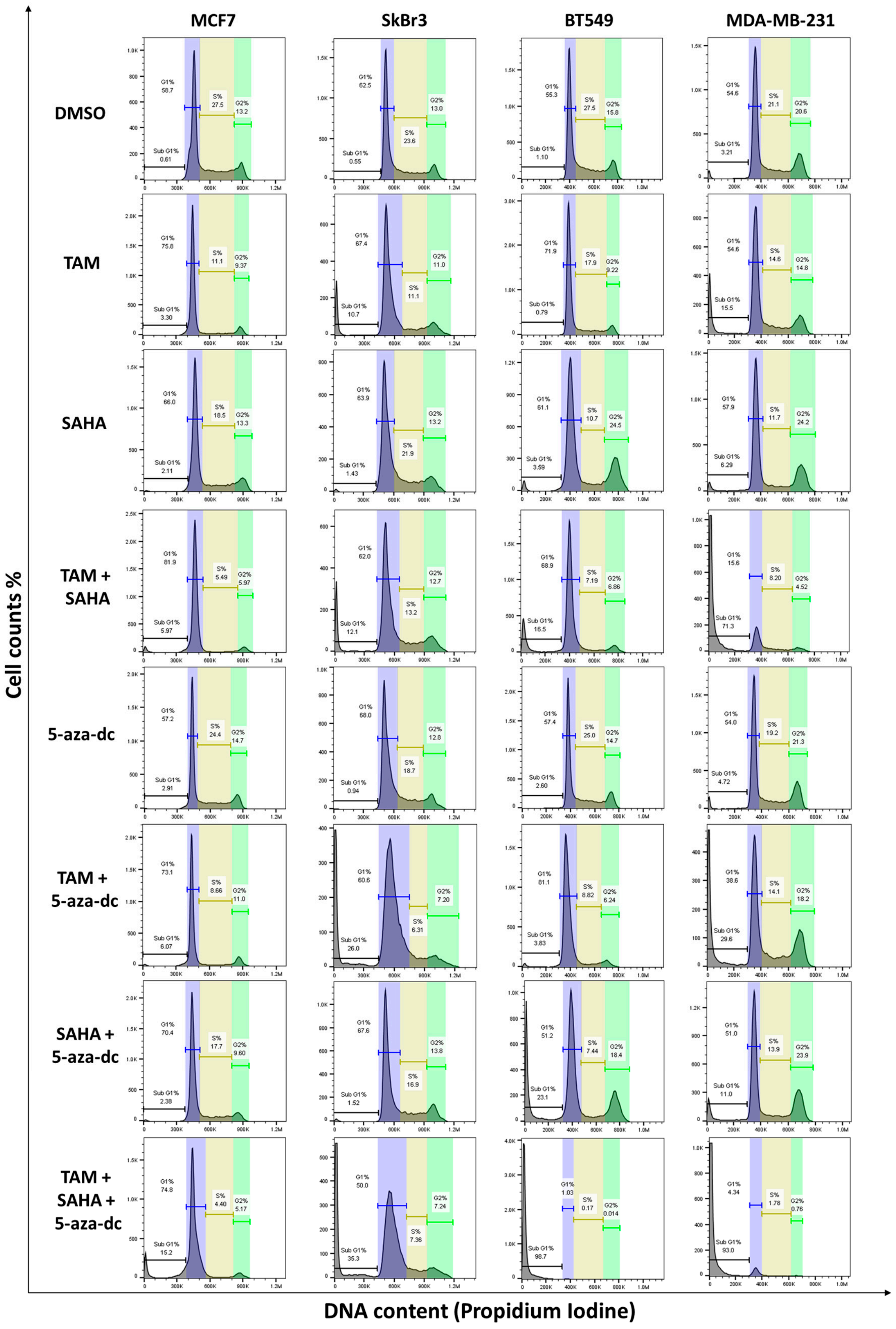

Figure S6. Effect of TAM, SAHA, 5-aza-dc and their combination on cell cycle distribution. Histograms represent cell cycle distribution of MCF7, SkBr3, BT-549 and MDA-MB-231 cells treated with IC50 concentrations of SAHA and/or 5-aza-dc and their combination with IC50 concentration of TAM. The X-axis is the DNA content (marked by PI), whereas the Y-axis is the cell number.

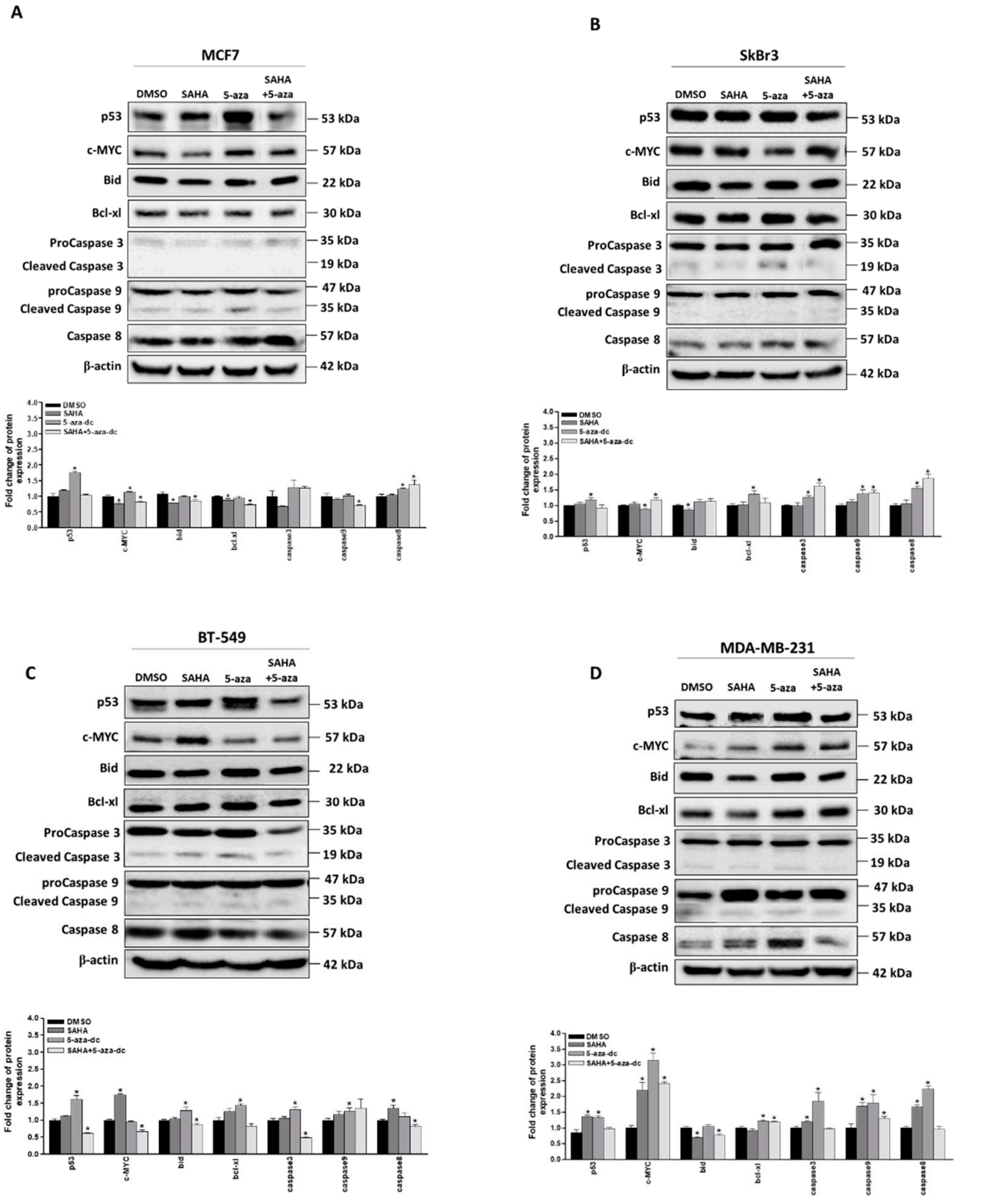

Figure S7. Effect of SAHA, 5-aza-dc and their combination on apoptosis in breast cancer cells. (**A**–**D**) Upper panels: Western blot analysis of the indicated proteins in (**A**) MCF7, (**B**) SkBr3, (**C**) BT-549 and (**D**) MDA-MB-231 cells after treatment with IC50 concentrations of SAHA and/or 5-aza-dc. β-actin protein was blotted as a loading control. Lower panels: Bar graphs showing relative fold changes of the indicated proteins after quantification and normalization to β-actin signal and DMSO treatment. Shown are the means ± SEM of at least three independent experiments. * $p < 0.05$ vs. DMSO group.
